# Evaluation of the Antigen mariPOC Respi Test Versus PCR in Relation to Immunological Viral Response in Children With Lower Respiratory Tract Infection

**DOI:** 10.1155/ijm/8832419

**Published:** 2025-10-15

**Authors:** Annika Eklundh, Samuel Rhedin, Ville Peltola, Matti Waris, Pontus Naucler, Giulia Gaudenzi, Alma Iacobelli, Magnus Lindh, Maria Andersson, Andreas Mårtensson, Tobias Alfvén, Malin Ryd-Rinder

**Affiliations:** ^1^Pediatric Emergency Unit, Sachs' Children and Youth Hospital, Stockholm, Sweden; ^2^Department of Global Public Health, Karolinska Institutet, Stockholm, Sweden; ^3^Department of Medical Epidemiology and Biostatistics, Karolinska Institutet, Stockholm, Sweden; ^4^Department of Pediatrics and Adolescent Medicine, Turku University Hospital and University of Turku, Turku, Finland; ^5^Institute of Biomedicine, University of Turku and Clinical Microbiology, Turku University Hospital, Turku, Finland; ^6^Division of Infectious Diseases, Department of Medicine, Karolinska Institutet, Stockholm, Sweden; ^7^Department of Infectious Diseases, Karolinska University Hospital, Solna, Sweden; ^8^Department of Infectious Diseases, University of Gothenburg, Gothenburg, Sweden; ^9^Global Health and Migration Unit, Department of Women's and Children's Health, Uppsala University, Uppsala, Sweden; ^10^Department of Infectious Diseases, Uppsala University Hospital, Uppsala, Sweden; ^11^Astrid Lindgren Children's Hospital, Karolinska University Hospital, Stockholm, Sweden; ^12^Department of Women's and Children's Health, Karolinska Institutet, Stockholm, Sweden

## Abstract

**Background:**

Real-time polymerase chain reaction (PCR), the gold standard for viral diagnostics in children, is a sensitive but resource-intensive method. Viral antigen tests are cheaper and more rapid but have lower sensitivity. The clinical relevance of PCR positivity has been questioned because of its high sensitivity and detection in asymptomatic individuals. Thus, we hypothesized that antigen test positivity might be more indicative of active infection than PCR positivity. The aim of this study was to evaluate the antigen test mariPOC Respi test for the detection of 10 respiratory viruses versus PCR in relation to viral load, days of illness, and immunological viral response.

**Methods:**

Children 1–59 months old with lower respiratory infections were prospectively enrolled at the emergency department, Sachs' Children and Youth Hospital, Stockholm, Sweden, between 2017 and 2019. Nasopharyngeal samples were collected from all cases (*n* = 314). The sensitivity and specificity of the mariPOC Respi test were assessed in children with and without an immunological viral response (defined as a blood myxovirus resistance Protein A level > 430 *μ*g/L), using PCR as the reference standard.

**Results:**

The highest sensitivity for mariPOC Respi test was attained for respiratory syncytial virus (68%; 95% confidence interval: 63–73). Restricting the analysis to cases with a viral immunological response did not alter the results considerably.

**Conclusion:**

These findings do not support the idea that mariPOC Respi test positivity to a higher degree than PCR correlates with clinical relevance, as indicated by an immunological viral response. The role of antigen tests in current clinical practice requires further discussion, particularly in the post–pandemic era.

**Trial Registration:**

ClinicalTrials.gov identifier: NCT03233516

## 1. Introduction

Respiratory infections in children are often of viral etiology [1, 2]. Real-time polymerase chain reaction (PCR) is a sensitive molecular-based method that is considered the gold standard for viral diagnostics in children [3]. However, the method's need for proximity to advanced laboratory equipment and high cost limit its usefulness for point-of-care decisions. There are several lateral flow-based point-of-care tests for antigen detection of the most common respiratory viruses on the market. In general, antigen tests are cheap, rapid, and easy to use but have reduced sensitivity compared to PCR. A test system with a broader panel of targets is the multianalyte point-of-care antigen detection test system (mariPOC Respi test) that detects 10 different respiratory viruses and one bacterium: Influenza A/B (Flu A/B), respiratory syncytial virus (RSV), adenovirus (AdV), bocavirus (HBoV), coronavirus OC43 (CoV), metapneumovirus (HMPV), parainfluenza Virus 1–3 (PIV), and *Streptococcus pneumoniae* (*S. pneumoniae*). The test provides a preliminary result of strongly positive samples after 20 min and a final result within 2 h (for low positive and negative samples) [4]. The sensitivity for RSV and Flu A/B has been reported to be as high as 90% compared to PCR, but the sensitivity for less common respiratory viruses such as HMPV, PIV 1–3, CoV OC43, and HBoV has not been sufficiently investigated [5, 6]. mariPOC Respi test can also detect SARS-CoV-2. However, because this study was conducted before the COVID-19 pandemic, this feature was not available at the time of the trial.

Although PCR is the gold standard for viral diagnostics, the high sensitivity of this method has raised concerns regarding the clinical relevance of test positivity, as viruses such as rhinovirus (RV), HBoV, and AdV are frequently detected in asymptomatic children [7, 8]. Thus, we hypothesized that antigen test positivity might be more indicative of an active infection than PCR positivity because the latter method is overly sensitive [9].

Assessments of the host immune response provide further information to address this diagnostic dilemma. Myxovirus resistance Protein A (MxA) is a novel viral biomarker involved in the interferon signaling pathway during the viral immunological response. Biomarker levels are lower in asymptomatic children than in symptomatic children who test positive for a respiratory virus by PCR [10]. Thus, the test shows promise in evaluating the significance of PCR results in specific situations.

The primary aim of this study was to evaluate the sensitivity and specificity of mariPOC Respi test versus PCR for the detection of 10 respiratory viruses. The secondary aim was to assess the performance of the mariPOC Respi test in relation to viral load, days of illness, and immunological viral response, using MxA as a marker for viral response.

## 2. Materials and Methods

### 2.1. Study Design and Study Setting

The study design of the Trial of Respiratory Infections in Children for Enhanced Diagnostics (TREND) study has been previously described [11]. The study was a hospital-based prospective observational study that took place at the emergency department of Sachs' Children and Youth Hospital, a tertiary care level hospital in Stockholm, Sweden, with approximately 30,000 visits each year. The study period was between November 2017 and December 2019, before the COVID-19 pandemic. The inclusion criteria were children aged 1–59 months with symptoms corresponding to clinical pneumonia according to the World Health Organization criteria: reported or observed breathing troubles/cough, age-adjusted tachypnea (≥ 50 breaths/min in children aged 1–12 months and ≥ 40/min in children aged > 1 year), or chest indrawing. Children could only be enrolled once and were excluded if they had been hospitalized during the previous 14 days [11]. Since the case definition resulted in the enrolment of a variety of acute respiratory syndromes and only a few children were diagnosed with pneumonia, we chose to use the term lower respiratory tract infection (LRTI) instead of pneumonia to describe the condition of the children [12].

One bacterium, *S. pneumoniae*, can be detected by the mariPOC Respi test; however, since this finding often represents colonization, the manufacturer does not recommend considering this result in children younger than 7 years. Hence, this result was not considered in the analysis.

### 2.2. Sampling and Data Collection

All samples were taken within 24 h of arrival at the emergency department, but almost always much quicker. A nasopharyngeal aspirate for PCR (diluted in 1.3 mL saline) and a nasopharyngeal swab for mariPOC Respi test (diluted in 1.3 mL mariPOC RTI buffer) were collected from all study subjects. Blood tests for MxA and C-reactive protein (CRP) were taken by finger prick. For the MxA analyses, 20 mL of capillary blood was collected using a heparinized plastic end-to-end capillary tube and then immediately diluted in a hypotonic in-house buffer. Samples that were not directly analyzed at the point of care (MxA blood sample and the nasopharyngeal aspirate) were stored at −80°C and then shipped batchwise on dry ice to minimize degradation of the sample material. All cases were examined by a physician, and the caregivers completed an electronic questionnaire with questions on background characteristics.

### 2.3. Microbiological and Biochemical Analyses

Analysis with the mariPOC Respi test was performed on the nasopharyngeal swabs directly at the emergency department of Sachs' Children and Youth Hospital at the time of enrolment, and the test results were available (but not always taken into consideration) to the treating physician.

The nasopharyngeal aspirates were analyzed using multiplex qPCR based on the TaqMan technique for Flu A/B, RSV A/B, AdV, HBoV, CoV (HKU1, NL63, OC43, and 229E), HMPV, PIV 1–3, RV, enterovirus (EV), *S. pneumoniae*, *Haemophilus influenzae*, *Bordetella pertussis*, *Mycoplasma pneumoniae*, and *Chlamydophila pneumoniae* at Clinical Microbiology, Sahlgrenska University Hospital, Gothenburg, using the ABI 7900 384-well system (Applied Biosystems, Foster City, California, United States) [13]. The nasopharyngeal aspirates were stored at −80°C before analysis. In this study, only pathogens that were also tested by mariPOC Respi were considered. PCR analyses were considered positive if the Ct value was ≤ 40, except for Flu B (Ct value ≤ 38) and HBoV (Ct value ≤ 36). The cutoff for Flu B was set to Ct ≤ 38, as clinical diagnostics sometimes observe noise in samples with Ct values between 38 and 40 (concluded as noise because rerunning with other assays have given negative results). The cutoff for HBoV was set to ≤ 36 based on our experience of frequently finding HBoV with Ct values between 36 and 40 as a coinfection when other (probably causative) pathogens have been identified.

CRP levels were analyzed at the point of care using an Alere Afinion AS100 Analyzer. MxA analyses were performed in batches at the Institute of Biomedicine, University of Turku, Finland, using an in-house enzyme immunoassay, which measures MxA levels against a recombinant human MxA standard produced in insect cells using baculovirus expression system. Interassay variation of the study runs was 18% [10].

### 2.4. Statistical Analyses

The performance of the mariPOC Respi test compared with qPCR was assessed by calculating the sensitivity, specificity, negative predictive value (NPV), and positive predictive value (PPV) for each virus. Data are presented as proportions (%), means with a 95% confidence interval (CI), or medians with interquartile range (IQR). An analysis was performed restricted to cases with an immunological response, defined as a blood MxA level > 430 *μ*g/L, which, according to a previous study, is an optimal cutoff to discriminate between viral and bacterial LRTI [12]. The test was also assessed in relation to viral load (using Ct values as a proxy for viral load, with a lower Ct value indicating a higher viral load) and days of illness. The Mann–Whitney *U* test was performed to determine if there was a significant difference in Ct values and MxA levels in the PCR-positive group between mariPOC-positive and mariPOC-negative cases, that is, a difference in viral load and immunological response. Data analysis was performed using Stata Version 16.1 (StataCorp, College Station, Texas, United States).

### 2.5. Ethics Statement

The study protocol, informed consent statement, clinical research form, and all other study documents were submitted to and approved by the Regional Ethics Committee of Stockholm (Dnr 2017/958-31). The caregivers provided written informed consent before inclusion in the study.

## 3. Results

### 3.1. Clinical and Laboratory Characteristics

After excluding 16 cases with missing mariPOC Respi data, 314 were included in the study. The sociodemographic and clinical characteristics of the cases were described previously [12, 14]. The median age was 13 months (IQR 6–22 months), and the median CRP level was 13 mg/L (IQR 0–33). The median number of days with respiratory tract symptoms before visiting the emergency department was 4 (IQR 2–6). With the mariPOC Respi test, 134/314 (43%) cases were positive for either AdV, HBoV, CoV, Flu A/B, HMPV, PIV 1–3, or RSV. Using PCR, 199/314 (63%) were positive for any of the same viruses (Table 1).

### 3.2. Sensitivity and Specificity for mariPOC Respi as Compared to PCR

The highest sensitivity for the mariPOC Respi test was attained for RSV (68%; 95% CI: 63–73), followed by Flu A/B (67%; 61–72). The specificities were high (96%–100%) for all included viruses. RSV was the most frequently detected virus (90/314 with the mariPOC Respi test and 122/314 with PCR). With the mariPOC Respi test, few cases were positive for Flu B (*n* = 2), HBoV (*n* = 3), PIV 1–3 (*n* = 3), and CoV (*n* = 4), which resulted in sensitivities as low as 20% or below, which is why these results are not presented in a table. For Flu A and B, a combined variable “Flu A/B” was created (Table 2).

### 3.3. Sensitivity and Specificity for mariPOC Respi in Relation to Immunological Response

The sensitivity and specificity of the mariPOC Respi test versus PCR were also evaluated in a subanalysis restricted to children with blood MxA levels > 430 *μ*g/L (*n* = 194). In this analysis, the highest sensitivity was attained for RSV (71%; 95% CI: 65–78), followed by Flu A/B (67%; 60–73) (Table 3). Overall, the sensitivities and specificities of the analyses restricted to cases with high MxA levels were similar to those of the main analysis.

### 3.4. Sensitivity and Specificity for mariPOC Respi in Relation to Duration of Symptoms

Finally, the sensitivity and specificity for mariPOC Respi versus PCR were evaluated with regard to the duration of symptoms of respiratory illness. In the group of children with symptoms for 1–4 days (*n* = 197), the highest sensitivity was seen for RSV (76%; 70–82): The sensitivity for RSV was lower in the group with symptoms for more than 4 days (57%; 48–66), whereas for Flu, the sensitivity was lower in the group with symptoms for 1–4 days (43%; 36–50) and higher in the other group (82%; 75–89) (Table 4).

### 3.5. Ct and MxA Values for PCR-Positive Cases, Divided Into mariPOC Positive and Negative

**Figure 1:**
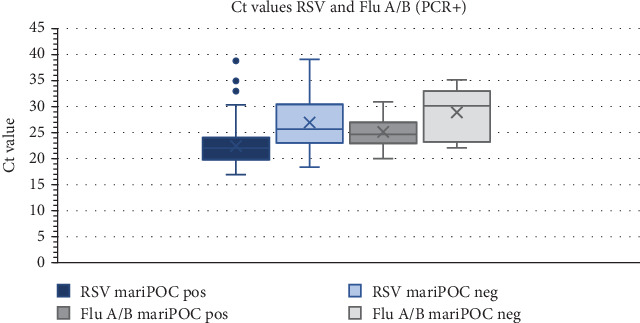
Ct values for RSV and Flu A/B. Boxplot of Ct values for all PCR-positive cases, divided into mariPOC-positive and mariPOC-negative cases. *p* values calculated with Mann–Whitney *U* test.

**Figure 2:**
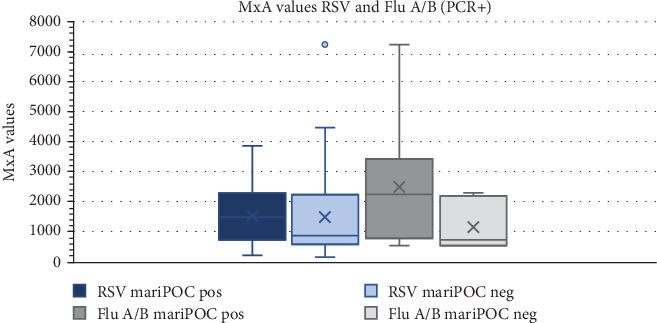
MxA values for RSV and Flu A/B. Boxplot of MxA values for all PCR-positive cases, divided into mariPOC-positive and mariPOC-negative cases. *p* values calculated with Mann–Whitney *U* test.

In the PCR-positive group, Ct values for RSV and Flu A/B were compared between mariPOC-positive and mariPOC-negative cases (false negative, since PCR positive) as an inverse approximation of viral load. Cases that were positive for RSV had significantly lower Ct values (*p* < 0.001) than false-negative cases. For Flu A/B, there was no significant difference between the groups (*p* > 0.05) (Figure 1). The MxA values were also compared between the groups, and no significant difference was observed for either RSV or Flu A/B (*p* > 0.05) (Figure 2).

## 4. Discussion

In this prospective study, we report that the mariPOC Respi antigen test showed low to moderate clinical sensitivity compared to PCR for the tested viruses in children under 5 years with acute LRTI and that the results were similar in children with and without a viral immunological response. To our knowledge, this is the first study to evaluate an antigen-based microbiological test in children while also considering the viral immunological response to account for PCR positivity without clinical relevance. However, restricting the analysis to cases with a viral immunological response, using MxA as a marker for viral response, did not alter the results considerably. Nevertheless, our study was not designed to evaluate the utility of including MxA in a diagnostic algorithm.

The highest sensitivity (68%) was observed for RSV, which is the most important respiratory pathogen in children [15]. The sensitivity for detecting HMPV was considerably lower than that for detecting RSV and Flu A/B (44%). For AdV, HBoV, CoV, and PIV 1–3, too few PCR-positive samples were detected to allow further evaluation.

We reported lower sensitivities for most viruses compared to previous studies, which could be attributed to several factors, such as mismatches between the test antibodies and circulating viruses, and that this study was performed on a narrower age interval compared to other studies on mariPOC Respi test [5, 6, 16, 17]. We used nasopharyngeal swabs to collect specimens, and a previous study showed that the sensitivity for mariPOC Respi test might be higher when nasopharyngeal aspirate sampling is used. However, as the nasopharyngeal aspirate sample requires centrifugation before mariPOC Respi analysis, it is less suitable for rapid point-of-care testing [5].

The specificity for mariPOC Respi test was high overall, which was consistent with previous studies [16, 17]. This means that, in general, a positive antigen test can be trusted. Thus, during the season when the infection rates are high, antigen testing could be used for rapid identification of positive cases with a high viral load. If complemented with PCR for the remaining cases, this could be a strategy that optimizes sensitivity, specificity, speed, and cost efficiency.

As the viral load differed over the course of the infection, we performed stratified analyses with regard to the duration of symptoms. For RSV, the highest sensitivity (76%; 70–82) was observed in the group of children with symptoms for 1–4 days. For Flu, the reverse was observed, where the sensitivity was higher in the group with symptoms for more than 4 days (82%; 75–89) [18]. This may reflect how viral load changes during the course of the disease.

Ivaska et al. performed a study in a pediatric emergency department, evaluating the accuracy of mariPOC Respi compared to qPCR for detecting respiratory viruses in symptomatic children. Nasopharyngeal samples (flocked swabs) were collected from 158 children (ages 0–16) with respiratory symptoms and/or fever, and the samples were analyzed by both mariPOC Respi and qPCR [16]. Comparing our results with those of this study, the overall diagnostic yield of mariPOC Respi for detecting any virus was similar (44% vs. 43%). Nevertheless, Ivaska et al.'s study reported higher sensitivities for RSV (89%), Flu A (71%), and Flu B (86%). Their study period was shorter (2 months) and performed during the peak of the respiratory infectious disease season compared to our 2-year study period, in which children were included during the entire year. Ivaska et al. also used stricter inclusion criteria (hospitalized with respiratory symptoms or fever without a focus or treated as outpatients with respiratory infections suspected to be caused by influenza virus, RSV, or AdV) with more severely ill children and children with symptoms corresponding to the tested viral agents [16].

Tuuminen et al. evaluated the mariPOC Respi test for the detection of Flu A and RSV by comparing nasopharyngeal aspirates with nasal swabs. In this study, nasal swabs generated lower sensitivities than aspirates did. The sensitivities for RSV and Flu A (in the same age group) were higher than those in our study; however, Tuuminen et al. used a direct fluorescent antibody assay as the primary reference method, which is less sensitive than PCR [5].

The clinical importance of viral PCR positivity for certain viruses has been questioned because of frequent asymptomatic detection [7, 18]. This challenge has become particularly evident during the COVID-19 pandemic when a substantial number of asymptomatic children have undergone viral testing. It has also been hypothesized that antigen test positivity is more clinically relevant than PCR positivity because PCR is overly sensitive [9]. To account for this, we performed a subanalysis restricted to children with a significant viral immune response, defined as elevated blood MxA levels that did not considerably alter the results. We also compared Ct values for RSV and Flu A/B in the PCR-positive group between mariPOC Respi–positive and mariPOC Respi–negative cases. For RSV, there was a significant difference between the groups, indicating that the mariPOC Respi–positive cases had a significantly higher viral load, which might suggest a more active infection. For Flu A/B, the number of cases was much smaller, and there was no significant difference between the groups. In a study by Jokela et al., the median Ct values for specimens with conflicting/nonmatching results (i.e., mariPOC negative and PCR positive) were significantly higher (30.4) compared to the specimens with matching results (mariPOC positive and PCR positive), 21.6 for real-time RT-PCR (*p* = 0.002) [19].

Although frequent asymptomatic detection of certain viruses through PCR might be a diagnostic challenge, our study does not support the idea that this is less of a problem in antigen-based tests, as is sometimes argued. The optimal MxA cutoff for differentiating symptomatic from asymptomatic infections has not been established, and there is a need for comprehensive studies that consider both the virological and immunological aspects of viral respiratory infections in children. It has been reported that RV infections elicit a lower MxA response than other viruses, and we used a higher MxA cutoff (430 *μ*g/L) than in previous studies on MxA (175 *μ*g/L, Toivonen et al; 200 ng/mL, Engelmann et al.) [10, 20]. This could have resulted in a different classification of some children with a low-grade viral immune response as having no viral immune response.

The strengths of this study were the well-defined cohort of children, which we believe can be generalized to similar pediatric emergency departments in Western countries, and the prospective and standardized collection of samples. The samples for PCR and mariPOC Respi test were collected and managed according to the manufacturer's instructions.

This study has some limitations. We were not able to study the effect of the mariPOC Respi test results on patient management/treatment because the results were not systematically given to the treating physician. Very few cases were positive for AdV (8), HBoV (3), CoV (4), and PIV 1–3 (3), which resulted in lower precision of the accuracy estimates. Furthermore, AdV, HBoV, and CoV are more often found in asymptomatic individuals than RSV and Flu, and there are not enough cases to study the correlation between viral response and the Ct value for these viruses, which would have been interesting [2].

Viral testing can be used for several purposes including diagnostic testing, assessment of infectiousness, and epidemiological purposes. How do antigen tests suit this context? From a clinical perspective, a sensitivity of 68% is insufficient in many situations, particularly when PCR in the post–pandemic era is readily available in hospital settings, at least in high-income countries.

With the exception of Flu, there is currently no specific antiviral treatment available for LRTIs, but diagnosing a viral infection can still be important in reducing the prescription of unnecessary antibiotics, although there are conflicting results on this topic [21, 22]. It is also crucial to remember that viral positivity does not rule out bacterial coinfection [22–24].

The use of PCR is limited by its excessive cost, bulky equipment, and need for separate laboratory facilities to prevent cross-contamination. mariPOC Respi analysis is more easily available than PCR and analyzes a wider selection of pathogens than lateral flow assays. Based on a low sensitivity but high specificity, antigen detection is best used for “ruling-in” infections (e.g., diagnostic testing and assessing infectiousness), rather than “ruling-out” (deciding on cohort care) or for epidemiological purposes. Hence, the mariPOC Respi test may have a role in outpatient settings during the epidemic season, focusing on a limited number of viruses, owing to its affordability and lack of need for a sophisticated laboratory setup.

## 5. Conclusion

In conclusion, this study provides additional insights into the performance of the mariPOC Respi antigen test system in young children with LRTI, indicating only moderate sensitivity for RSV and Flu A/B and low sensitivity for all other viruses. Using MxA as a marker of immunological viral response, we could not verify the hypothesis that the relatively lower sensitivity of mariPOC could be explained by a stronger association with clinical infection or immunological response.

The role of antigen tests in current clinical practice in the post–pandemic era requires further discussion.

## Figures and Tables

**Table 1:** Demographic, clinical, and laboratory characteristics of study subjects (*n* = 314).

**Characteristic**	**n** **(%)**
Age (months), median (IQR)	13 (6–22)
1–11 months	144 (46)
12–59 months	170 (54)
Duration of symptoms (days), median (IQR)	4 (2–6)
Symptoms for 1–4 days	197 (63)
Symptoms for more than 4 days	115 (37)
C-reactive protein (mg/L), median (IQR)	13 (0–33)
> 80	28 (9)
> 120	10 (3)
Myxovirus resistance Protein A (*μ*g/L), median (IQR)	668 (245–1464)
≤ 430	120 (38)
> 430	194 (62)
Samples positive/negative for mariPOC	134 (43)/180 (57)
Samples positive/negative for PCR^a^	199 (63)/115 (37)

*Note:* Enterovirus and rhinoviruses were excluded because they are not included in mariPOC.

^a^PCR analyses for AdV, HBoV, CoV, Flu A/B, HMPV, RSV, and PIV.

**Table 2:** Sensitivities, specificities, PPV, and NPV of mariPOC compared to qPCR (*n* = 314).

**Virus**	**mariPOC**	**qPCR**		**% (95% confidence interval)**			
	**(** **n** ** = pos/neg)**	**+**	**−**	**Sensitivity**	**Specificity**	**PPV**	**NPV**
RSV	+ (90)	83	7	68 (63–73)	96 (94–98)	92 (89–95)	83 (78–87)
	− (224)	39	185				
Flu A	+ (14)	10	4	67 (61–72)	99 (97–99)	71 (66–76)	98 (97–100)
	− (299)	5	294				
Flu A/B^a^	+ (16)	12	4	67 (61–72)	99 (97–100)	75 (70–80)	98 (96–100)
	− (298)	6	292				
HMPV	+ (16)	14	2	44 (38–49)	99 (98–100)	88 (84–91)	94 (91–97)
	− (298)	18	280				

^a^Since there were few cases positive for Flu B (*n* = 2), a combined variable was created—Flu A/B.

**Table 3:** Sensitivities, specificities, PPV, and NPV of mariPOC compared to qPCR for patients with MxA levels > 430 *μ*g/L (*n* = 194).

**Virus**	**mariPOC**	**qPCR**		**% (95% confidence interval)**			
	**(** **n** ** = pos/neg)**	**+**	**−**	**Sensitivity**	**Specificity**	**PPV**	**NPV**
RSV	+ (84)	79	5	71 (65–78)	94 (91–97)	94 (91–97)	71 (65–77)
	− (110)	32	78				
Flu A/B	+ (16)	12	4	67 (60–73)	98 (96–100)	75 (69–81)	97 (94–99)
	− (178)	6	172				
HMPV	+ (16)	14	2	45 (38–52)	99 (97–100)	88 (83–92)	90 (86–95)
	− (178)	17	161				

**Table 4:** Comparison of sensitivities and specificities of mariPOC compared to qPCR in children with different durations of symptoms of respiratory illness.

**Duration of symptoms**								
	**1–4 days (** **n** = 197**)**				**More than 4 days (** **n** = 115**)**			
**Virus**	**n**	**mP +/−**	**Sensitivity**	**Specificity**	**n**	**mP +/−**	**Sensitivity**	**Specificity**
RSV	197	55/142	76 (70–82)	97 (95–99)	115	34/81	57 (48–66)	95 (91–99)
Flu A/B	197	5/192	43 (36–50)	99 (98–100)	115	11/104	82 (75–89)	98 (96–101)
HMPV	197	11/186	56 (49–62)	99 (98–100)	115	5/110	29 (20–37)	99 (97–101)

Abbreviation: mP, mariPOC.

## Data Availability

Deidentified participant data can be provided upon request if providing a reasonable proposal once an appropriate data-sharing agreement has been established with Karolinska Institutet.
